# Supplementary material for: Overlapping cell population expression profiling and regulatory inference in C. elegans
Source: BMC Genomics. 2016 Feb 29;17:159. doi: 10.1186/s12864-016-2482-z (PMC4772325; doi:10.1186/s12864-016-2482-z)
Supplement: Additional file 13: — Web supplement. (DOC 21 kb) [file 12864_2016_2482_MOESM13_ESM.zip › sortWeb/clusters/hier.300.clusters/44.html]

Cluster 44 

## Cluster 44

### Expression

| cnd-1 rep. 1 | cnd-1 rep. 2 | cnd-1 rep. 3 | pha-4 rep. 1 | pha-4 rep. 2 | pha-4 rep. 3 | ceh-27 | ceh-36 | ceh-6 | F21D5.9 | mir-57 | mls-2 | pal-1 | pros-1 | ttx-3 | unc-130 | hlh-16 | irx-1 | ceh-6 (+) hlh-16 (+) | ceh-6 (+) hlh-16 (-) | ceh-6 (-) hlh-16 (+) | cnd-1 singlets | pha-4 singlets | 0 | 60 | 120 | 150 | 180 | 240 | 330 | 390 | 420 | 480 | 540 | 570 | 600 | 630 | 660 | NAME | Functional description |
| --- | --- | --- | --- | --- | --- | --- | --- | --- | --- | --- | --- | --- | --- | --- | --- | --- | --- | --- | --- | --- | --- | --- | --- | --- | --- | --- | --- | --- | --- | --- | --- | --- | --- | --- | --- | --- | --- | --- | --- |
|  |  |  |  |  |  |  |  |  |  |  |  |  |  |  |  |  |  |  |  |  |  |  |  |  |  |  |  |  |  |  |  |  |  |  |  |  |  | *cdd-2* | CytiDine Deaminase |
|  |  |  |  |  |  |  |  |  |  |  |  |  |  |  |  |  |  |  |  |  |  |  |  |  |  |  |  |  |  |  |  |  |  |  |  |  |  | F46C8.16 |  |
|  |  |  |  |  |  |  |  |  |  |  |  |  |  |  |  |  |  |  |  |  |  |  |  |  |  |  |  |  |  |  |  |  |  |  |  |  |  | *sre-33* | Serpentine Receptor, class E (epsilon) |
|  |  |  |  |  |  |  |  |  |  |  |  |  |  |  |  |  |  |  |  |  |  |  |  |  |  |  |  |  |  |  |  |  |  |  |  |  |  | *chil-16* | CHItinase-Like |
|  |  |  |  |  |  |  |  |  |  |  |  |  |  |  |  |  |  |  |  |  |  |  |  |  |  |  |  |  |  |  |  |  |  |  |  |  |  | *srh-42* | Serpentine Receptor, class H |
|  |  |  |  |  |  |  |  |  |  |  |  |  |  |  |  |  |  |  |  |  |  |  |  |  |  |  |  |  |  |  |  |  |  |  |  |  |  | *srj-6* | Serpentine Receptor, class J |
|  |  |  |  |  |  |  |  |  |  |  |  |  |  |  |  |  |  |  |  |  |  |  |  |  |  |  |  |  |  |  |  |  |  |  |  |  |  | *srv-8* | Serpentine Receptor, class V |
|  |  |  |  |  |  |  |  |  |  |  |  |  |  |  |  |  |  |  |  |  |  |  |  |  |  |  |  |  |  |  |  |  |  |  |  |  |  | C08E3.13 |  |
|  |  |  |  |  |  |  |  |  |  |  |  |  |  |  |  |  |  |  |  |  |  |  |  |  |  |  |  |  |  |  |  |  |  |  |  |  |  | M02D8.2 |  |
|  |  |  |  |  |  |  |  |  |  |  |  |  |  |  |  |  |  |  |  |  |  |  |  |  |  |  |  |  |  |  |  |  |  |  |  |  |  | Y102A5C.36 |  |
|  |  |  |  |  |  |  |  |  |  |  |  |  |  |  |  |  |  |  |  |  |  |  |  |  |  |  |  |  |  |  |  |  |  |  |  |  |  | *srh-180* | Serpentine Receptor, class H |
|  |  |  |  |  |  |  |  |  |  |  |  |  |  |  |  |  |  |  |  |  |  |  |  |  |  |  |  |  |  |  |  |  |  |  |  |  |  | *med-1* | Mesoderm and Endoderm Determination |
|  |  |  |  |  |  |  |  |  |  |  |  |  |  |  |  |  |  |  |  |  |  |  |  |  |  |  |  |  |  |  |  |  |  |  |  |  |  | F35D11.5 |  |
|  |  |  |  |  |  |  |  |  |  |  |  |  |  |  |  |  |  |  |  |  |  |  |  |  |  |  |  |  |  |  |  |  |  |  |  |  |  | *lact-6* | beta-LACTamase domain containing |
|  |  |  |  |  |  |  |  |  |  |  |  |  |  |  |  |  |  |  |  |  |  |  |  |  |  |  |  |  |  |  |  |  |  |  |  |  |  | F45H11.10 |  |
|  |  |  |  |  |  |  |  |  |  |  |  |  |  |  |  |  |  |  |  |  |  |  |  |  |  |  |  |  |  |  |  |  |  |  |  |  |  | R10H1.7 |  |
|  |  |  |  |  |  |  |  |  |  |  |  |  |  |  |  |  |  |  |  |  |  |  |  |  |  |  |  |  |  |  |  |  |  |  |  |  |  | C26C6.8 |  |
|  |  |  |  |  |  |  |  |  |  |  |  |  |  |  |  |  |  |  |  |  |  |  |  |  |  |  |  |  |  |  |  |  |  |  |  |  |  | *grl-11* | GRound-Like (grd related) |
|  |  |  |  |  |  |  |  |  |  |  |  |  |  |  |  |  |  |  |  |  |  |  |  |  |  |  |  |  |  |  |  |  |  |  |  |  |  | C01G12.5 |  |
|  |  |  |  |  |  |  |  |  |  |  |  |  |  |  |  |  |  |  |  |  |  |  |  |  |  |  |  |  |  |  |  |  |  |  |  |  |  | *cal-3* | CALmodulin related genes |
|  |  |  |  |  |  |  |  |  |  |  |  |  |  |  |  |  |  |  |  |  |  |  |  |  |  |  |  |  |  |  |  |  |  |  |  |  |  | *cgr-1* | CRAL/TRIO and GOLD domain suppressor of activated Ras |
|  |  |  |  |  |  |  |  |  |  |  |  |  |  |  |  |  |  |  |  |  |  |  |  |  |  |  |  |  |  |  |  |  |  |  |  |  |  | Y4C6B.4 |  |
|  |  |  |  |  |  |  |  |  |  |  |  |  |  |  |  |  |  |  |  |  |  |  |  |  |  |  |  |  |  |  |  |  |  |  |  |  |  | *degt-1* | DEGenerin-related Touch-involved channel |
|  |  |  |  |  |  |  |  |  |  |  |  |  |  |  |  |  |  |  |  |  |  |  |  |  |  |  |  |  |  |  |  |  |  |  |  |  |  | C17D12.3 |  |
|  |  |  |  |  |  |  |  |  |  |  |  |  |  |  |  |  |  |  |  |  |  |  |  |  |  |  |  |  |  |  |  |  |  |  |  |  |  | W10C8.6 |  |
|  |  |  |  |  |  |  |  |  |  |  |  |  |  |  |  |  |  |  |  |  |  |  |  |  |  |  |  |  |  |  |  |  |  |  |  |  |  | F29B9.5 |  |
|  |  |  |  |  |  |  |  |  |  |  |  |  |  |  |  |  |  |  |  |  |  |  |  |  |  |  |  |  |  |  |  |  |  |  |  |  |  | *sru-39* | Serpentine Receptor, class U |
|  |  |  |  |  |  |  |  |  |  |  |  |  |  |  |  |  |  |  |  |  |  |  |  |  |  |  |  |  |  |  |  |  |  |  |  |  |  | T10B9.9 |  |
|  |  |  |  |  |  |  |  |  |  |  |  |  |  |  |  |  |  |  |  |  |  |  |  |  |  |  |  |  |  |  |  |  |  |  |  |  |  | Y75B8A.11 |  |
|  |  |  |  |  |  |  |  |  |  |  |  |  |  |  |  |  |  |  |  |  |  |  |  |  |  |  |  |  |  |  |  |  |  |  |  |  |  | C30H6.10 |  |
|  |  |  |  |  |  |  |  |  |  |  |  |  |  |  |  |  |  |  |  |  |  |  |  |  |  |  |  |  |  |  |  |  |  |  |  |  |  | ZK813.9 |  |
|  |  |  |  |  |  |  |  |  |  |  |  |  |  |  |  |  |  |  |  |  |  |  |  |  |  |  |  |  |  |  |  |  |  |  |  |  |  | *srb-16* | Serpentine Receptor, class B (beta) |
|  |  |  |  |  |  |  |  |  |  |  |  |  |  |  |  |  |  |  |  |  |  |  |  |  |  |  |  |  |  |  |  |  |  |  |  |  |  | *lgc-29* | Ligand-Gated ion Channel |
|  |  |  |  |  |  |  |  |  |  |  |  |  |  |  |  |  |  |  |  |  |  |  |  |  |  |  |  |  |  |  |  |  |  |  |  |  |  | *gur-3* | GUstatory Receptor family |
|  |  |  |  |  |  |  |  |  |  |  |  |  |  |  |  |  |  |  |  |  |  |  |  |  |  |  |  |  |  |  |  |  |  |  |  |  |  | F59B10.3 |  |
|  |  |  |  |  |  |  |  |  |  |  |  |  |  |  |  |  |  |  |  |  |  |  |  |  |  |  |  |  |  |  |  |  |  |  |  |  |  | *ceh-19* | C. Elegans Homeobox |
|  |  |  |  |  |  |  |  |  |  |  |  |  |  |  |  |  |  |  |  |  |  |  |  |  |  |  |  |  |  |  |  |  |  |  |  |  |  | *nhr-195* | Nuclear Hormone Receptor family |
|  |  |  |  |  |  |  |  |  |  |  |  |  |  |  |  |  |  |  |  |  |  |  |  |  |  |  |  |  |  |  |  |  |  |  |  |  |  | *sup-1* | SUPpressor |
|  |  |  |  |  |  |  |  |  |  |  |  |  |  |  |  |  |  |  |  |  |  |  |  |  |  |  |  |  |  |  |  |  |  |  |  |  |  | *magi-1* | MAGI (Membrane Associated Guanylate kinase Inverted) homolog |
|  |  |  |  |  |  |  |  |  |  |  |  |  |  |  |  |  |  |  |  |  |  |  |  |  |  |  |  |  |  |  |  |  |  |  |  |  |  | *dpf-2* | Dipeptidyl Peptidase Four (IV) family |
|  |  |  |  |  |  |  |  |  |  |  |  |  |  |  |  |  |  |  |  |  |  |  |  |  |  |  |  |  |  |  |  |  |  |  |  |  |  | *nhr-21* | Nuclear Hormone Receptor family |
|  |  |  |  |  |  |  |  |  |  |  |  |  |  |  |  |  |  |  |  |  |  |  |  |  |  |  |  |  |  |  |  |  |  |  |  |  |  | C05D10.4 |  |
|  |  |  |  |  |  |  |  |  |  |  |  |  |  |  |  |  |  |  |  |  |  |  |  |  |  |  |  |  |  |  |  |  |  |  |  |  |  | D1007.19 |  |
|  |  |  |  |  |  |  |  |  |  |  |  |  |  |  |  |  |  |  |  |  |  |  |  |  |  |  |  |  |  |  |  |  |  |  |  |  |  | K07A12.8 |  |
|  |  |  |  |  |  |  |  |  |  |  |  |  |  |  |  |  |  |  |  |  |  |  |  |  |  |  |  |  |  |  |  |  |  |  |  |  |  | K07C10.3 |  |
|  |  |  |  |  |  |  |  |  |  |  |  |  |  |  |  |  |  |  |  |  |  |  |  |  |  |  |  |  |  |  |  |  |  |  |  |  |  | R13H4.7 |  |
|  |  |  |  |  |  |  |  |  |  |  |  |  |  |  |  |  |  |  |  |  |  |  |  |  |  |  |  |  |  |  |  |  |  |  |  |  |  | H03E18.2 |  |
|  |  |  |  |  |  |  |  |  |  |  |  |  |  |  |  |  |  |  |  |  |  |  |  |  |  |  |  |  |  |  |  |  |  |  |  |  |  | ZK757.1 |  |
|  |  |  |  |  |  |  |  |  |  |  |  |  |  |  |  |  |  |  |  |  |  |  |  |  |  |  |  |  |  |  |  |  |  |  |  |  |  | C50B8.4 |  |
|  |  |  |  |  |  |  |  |  |  |  |  |  |  |  |  |  |  |  |  |  |  |  |  |  |  |  |  |  |  |  |  |  |  |  |  |  |  | C51E3.10 |  |
|  |  |  |  |  |  |  |  |  |  |  |  |  |  |  |  |  |  |  |  |  |  |  |  |  |  |  |  |  |  |  |  |  |  |  |  |  |  | *npr-6* | NeuroPeptide Receptor family |
|  |  |  |  |  |  |  |  |  |  |  |  |  |  |  |  |  |  |  |  |  |  |  |  |  |  |  |  |  |  |  |  |  |  |  |  |  |  | *ckb-4* | Choline Kinase B |
|  |  |  |  |  |  |  |  |  |  |  |  |  |  |  |  |  |  |  |  |  |  |  |  |  |  |  |  |  |  |  |  |  |  |  |  |  |  | C05D11.13 |  |
|  |  |  |  |  |  |  |  |  |  |  |  |  |  |  |  |  |  |  |  |  |  |  |  |  |  |  |  |  |  |  |  |  |  |  |  |  |  | T02E9.5 |  |
|  |  |  |  |  |  |  |  |  |  |  |  |  |  |  |  |  |  |  |  |  |  |  |  |  |  |  |  |  |  |  |  |  |  |  |  |  |  | F22B5.4 |  |
|  |  |  |  |  |  |  |  |  |  |  |  |  |  |  |  |  |  |  |  |  |  |  |  |  |  |  |  |  |  |  |  |  |  |  |  |  |  | *wht-1* | WHiTe (Drosophila) related ABC transporter |
|  |  |  |  |  |  |  |  |  |  |  |  |  |  |  |  |  |  |  |  |  |  |  |  |  |  |  |  |  |  |  |  |  |  |  |  |  |  | K03E5.2 |  |
|  |  |  |  |  |  |  |  |  |  |  |  |  |  |  |  |  |  |  |  |  |  |  |  |  |  |  |  |  |  |  |  |  |  |  |  |  |  | ZK265.7 |  |
|  |  |  |  |  |  |  |  |  |  |  |  |  |  |  |  |  |  |  |  |  |  |  |  |  |  |  |  |  |  |  |  |  |  |  |  |  |  | *prmn-1* | PRoMiNin (5-transmembrane domain glycoprotein) homolog |
|  |  |  |  |  |  |  |  |  |  |  |  |  |  |  |  |  |  |  |  |  |  |  |  |  |  |  |  |  |  |  |  |  |  |  |  |  |  | T28B4.4 |  |
|  |  |  |  |  |  |  |  |  |  |  |  |  |  |  |  |  |  |  |  |  |  |  |  |  |  |  |  |  |  |  |  |  |  |  |  |  |  | F31E3.2 |  |
|  |  |  |  |  |  |  |  |  |  |  |  |  |  |  |  |  |  |  |  |  |  |  |  |  |  |  |  |  |  |  |  |  |  |  |  |  |  | ZC328.3 |  |
|  |  |  |  |  |  |  |  |  |  |  |  |  |  |  |  |  |  |  |  |  |  |  |  |  |  |  |  |  |  |  |  |  |  |  |  |  |  | C30F12.1 |  |
|  |  |  |  |  |  |  |  |  |  |  |  |  |  |  |  |  |  |  |  |  |  |  |  |  |  |  |  |  |  |  |  |  |  |  |  |  |  | *sdpn-1* | SynDaPiN (synaptic dynamin binding protein) homolog |
|  |  |  |  |  |  |  |  |  |  |  |  |  |  |  |  |  |  |  |  |  |  |  |  |  |  |  |  |  |  |  |  |  |  |  |  |  |  | *cka-2* | Choline Kinase A |
|  |  |  |  |  |  |  |  |  |  |  |  |  |  |  |  |  |  |  |  |  |  |  |  |  |  |  |  |  |  |  |  |  |  |  |  |  |  | C32D5.7 |  |
|  |  |  |  |  |  |  |  |  |  |  |  |  |  |  |  |  |  |  |  |  |  |  |  |  |  |  |  |  |  |  |  |  |  |  |  |  |  | F48A9.1 |  |
|  |  |  |  |  |  |  |  |  |  |  |  |  |  |  |  |  |  |  |  |  |  |  |  |  |  |  |  |  |  |  |  |  |  |  |  |  |  | *cgt-1* | Ceramide Glucosyl Transferase |
|  |  |  |  |  |  |  |  |  |  |  |  |  |  |  |  |  |  |  |  |  |  |  |  |  |  |  |  |  |  |  |  |  |  |  |  |  |  | F48A9.2 |  |
|  |  |  |  |  |  |  |  |  |  |  |  |  |  |  |  |  |  |  |  |  |  |  |  |  |  |  |  |  |  |  |  |  |  |  |  |  |  | F20A1.4 |  |
|  |  |  |  |  |  |  |  |  |  |  |  |  |  |  |  |  |  |  |  |  |  |  |  |  |  |  |  |  |  |  |  |  |  |  |  |  |  | T05E12.9 |  |
|  |  |  |  |  |  |  |  |  |  |  |  |  |  |  |  |  |  |  |  |  |  |  |  |  |  |  |  |  |  |  |  |  |  |  |  |  |  | *rab-1* | RAB family |
|  |  |  |  |  |  |  |  |  |  |  |  |  |  |  |  |  |  |  |  |  |  |  |  |  |  |  |  |  |  |  |  |  |  |  |  |  |  | *sre-3* | Serpentine Receptor, class E (epsilon) |
|  |  |  |  |  |  |  |  |  |  |  |  |  |  |  |  |  |  |  |  |  |  |  |  |  |  |  |  |  |  |  |  |  |  |  |  |  |  | *npr-21* | NeuroPeptide Receptor family |
|  |  |  |  |  |  |  |  |  |  |  |  |  |  |  |  |  |  |  |  |  |  |  |  |  |  |  |  |  |  |  |  |  |  |  |  |  |  | *tag-272* | Temporarily Assigned Gene name |
|  |  |  |  |  |  |  |  |  |  |  |  |  |  |  |  |  |  |  |  |  |  |  |  |  |  |  |  |  |  |  |  |  |  |  |  |  |  | F54H12.8 |  |
|  |  |  |  |  |  |  |  |  |  |  |  |  |  |  |  |  |  |  |  |  |  |  |  |  |  |  |  |  |  |  |  |  |  |  |  |  |  | *drag-1* | DRAGon (zebra fish) homolog |
|  |  |  |  |  |  |  |  |  |  |  |  |  |  |  |  |  |  |  |  |  |  |  |  |  |  |  |  |  |  |  |  |  |  |  |  |  |  | C35A5.10 |  |
|  |  |  |  |  |  |  |  |  |  |  |  |  |  |  |  |  |  |  |  |  |  |  |  |  |  |  |  |  |  |  |  |  |  |  |  |  |  | C25E10.10 |  |
|  |  |  |  |  |  |  |  |  |  |  |  |  |  |  |  |  |  |  |  |  |  |  |  |  |  |  |  |  |  |  |  |  |  |  |  |  |  | *nhr-186* | Nuclear Hormone Receptor family |
|  |  |  |  |  |  |  |  |  |  |  |  |  |  |  |  |  |  |  |  |  |  |  |  |  |  |  |  |  |  |  |  |  |  |  |  |  |  | *tag-253* | Temporarily Assigned Gene name |
|  |  |  |  |  |  |  |  |  |  |  |  |  |  |  |  |  |  |  |  |  |  |  |  |  |  |  |  |  |  |  |  |  |  |  |  |  |  | Y45G5AM.9 |  |
|  |  |  |  |  |  |  |  |  |  |  |  |  |  |  |  |  |  |  |  |  |  |  |  |  |  |  |  |  |  |  |  |  |  |  |  |  |  | *pqn-51* | Prion-like-(Q/N-rich)-domain-bearing protein |
|  |  |  |  |  |  |  |  |  |  |  |  |  |  |  |  |  |  |  |  |  |  |  |  |  |  |  |  |  |  |  |  |  |  |  |  |  |  | *mdt-15* | MeDiaTor |
|  |  |  |  |  |  |  |  |  |  |  |  |  |  |  |  |  |  |  |  |  |  |  |  |  |  |  |  |  |  |  |  |  |  |  |  |  |  | *nhr-46* | Nuclear Hormone Receptor family |
|  |  |  |  |  |  |  |  |  |  |  |  |  |  |  |  |  |  |  |  |  |  |  |  |  |  |  |  |  |  |  |  |  |  |  |  |  |  | *igcm-1* | ImmunoGlobulin-like Cell adhesion Molecule family |
|  |  |  |  |  |  |  |  |  |  |  |  |  |  |  |  |  |  |  |  |  |  |  |  |  |  |  |  |  |  |  |  |  |  |  |  |  |  | Y64G10A.6 |  |
|  |  |  |  |  |  |  |  |  |  |  |  |  |  |  |  |  |  |  |  |  |  |  |  |  |  |  |  |  |  |  |  |  |  |  |  |  |  | *sid-3* | Systemic RNA Interference Defective |
|  |  |  |  |  |  |  |  |  |  |  |  |  |  |  |  |  |  |  |  |  |  |  |  |  |  |  |  |  |  |  |  |  |  |  |  |  |  | *frpr-10* | FMRFamide Peptide Receptor family |
|  |  |  |  |  |  |  |  |  |  |  |  |  |  |  |  |  |  |  |  |  |  |  |  |  |  |  |  |  |  |  |  |  |  |  |  |  |  | *nhr-118* | Nuclear Hormone Receptor family |
|  |  |  |  |  |  |  |  |  |  |  |  |  |  |  |  |  |  |  |  |  |  |  |  |  |  |  |  |  |  |  |  |  |  |  |  |  |  | T05E12.8 |  |
|  |  |  |  |  |  |  |  |  |  |  |  |  |  |  |  |  |  |  |  |  |  |  |  |  |  |  |  |  |  |  |  |  |  |  |  |  |  | BE0003N10.t2 |  |
|  |  |  |  |  |  |  |  |  |  |  |  |  |  |  |  |  |  |  |  |  |  |  |  |  |  |  |  |  |  |  |  |  |  |  |  |  |  | C10A4.11 |  |
|  |  |  |  |  |  |  |  |  |  |  |  |  |  |  |  |  |  |  |  |  |  |  |  |  |  |  |  |  |  |  |  |  |  |  |  |  |  | ZK131.11 |  |
|  |  |  |  |  |  |  |  |  |  |  |  |  |  |  |  |  |  |  |  |  |  |  |  |  |  |  |  |  |  |  |  |  |  |  |  |  |  | C17H1.13 |  |
|  |  |  |  |  |  |  |  |  |  |  |  |  |  |  |  |  |  |  |  |  |  |  |  |  |  |  |  |  |  |  |  |  |  |  |  |  |  | F54D12.1 |  |
|  |  |  |  |  |  |  |  |  |  |  |  |  |  |  |  |  |  |  |  |  |  |  |  |  |  |  |  |  |  |  |  |  |  |  |  |  |  | T14F9.18 |  |
|  |  |  |  |  |  |  |  |  |  |  |  |  |  |  |  |  |  |  |  |  |  |  |  |  |  |  |  |  |  |  |  |  |  |  |  |  |  | C25A11.8 |  |
|  |  |  |  |  |  |  |  |  |  |  |  |  |  |  |  |  |  |  |  |  |  |  |  |  |  |  |  |  |  |  |  |  |  |  |  |  |  | *spsb-1* | SPSB (SPry and Socs Box) family |
|  |  |  |  |  |  |  |  |  |  |  |  |  |  |  |  |  |  |  |  |  |  |  |  |  |  |  |  |  |  |  |  |  |  |  |  |  |  | Y51H7C.8 |  |
|  |  |  |  |  |  |  |  |  |  |  |  |  |  |  |  |  |  |  |  |  |  |  |  |  |  |  |  |  |  |  |  |  |  |  |  |  |  | F43D2.6 |  |
|  |  |  |  |  |  |  |  |  |  |  |  |  |  |  |  |  |  |  |  |  |  |  |  |  |  |  |  |  |  |  |  |  |  |  |  |  |  | F58E6.4 |  |
|  |  |  |  |  |  |  |  |  |  |  |  |  |  |  |  |  |  |  |  |  |  |  |  |  |  |  |  |  |  |  |  |  |  |  |  |  |  | *srh-207* | Serpentine Receptor, class H |
|  |  |  |  |  |  |  |  |  |  |  |  |  |  |  |  |  |  |  |  |  |  |  |  |  |  |  |  |  |  |  |  |  |  |  |  |  |  | T13H10.8 |  |
|  |  |  |  |  |  |  |  |  |  |  |  |  |  |  |  |  |  |  |  |  |  |  |  |  |  |  |  |  |  |  |  |  |  |  |  |  |  | R13F6.13 |  |
|  |  |  |  |  |  |  |  |  |  |  |  |  |  |  |  |  |  |  |  |  |  |  |  |  |  |  |  |  |  |  |  |  |  |  |  |  |  | C51E3.8 |  |
|  |  |  |  |  |  |  |  |  |  |  |  |  |  |  |  |  |  |  |  |  |  |  |  |  |  |  |  |  |  |  |  |  |  |  |  |  |  | Y70C5C.8 |  |
|  |  |  |  |  |  |  |  |  |  |  |  |  |  |  |  |  |  |  |  |  |  |  |  |  |  |  |  |  |  |  |  |  |  |  |  |  |  | *clec-21* | C-type LECtin |
|  |  |  |  |  |  |  |  |  |  |  |  |  |  |  |  |  |  |  |  |  |  |  |  |  |  |  |  |  |  |  |  |  |  |  |  |  |  | F54B11.13 |  |
|  |  |  |  |  |  |  |  |  |  |  |  |  |  |  |  |  |  |  |  |  |  |  |  |  |  |  |  |  |  |  |  |  |  |  |  |  |  | F54H12.7 |  |
|  |  |  |  |  |  |  |  |  |  |  |  |  |  |  |  |  |  |  |  |  |  |  |  |  |  |  |  |  |  |  |  |  |  |  |  |  |  | F15H10.7 |  |
|  |  |  |  |  |  |  |  |  |  |  |  |  |  |  |  |  |  |  |  |  |  |  |  |  |  |  |  |  |  |  |  |  |  |  |  |  |  | M01E11.9 |  |
|  |  |  |  |  |  |  |  |  |  |  |  |  |  |  |  |  |  |  |  |  |  |  |  |  |  |  |  |  |  |  |  |  |  |  |  |  |  | T14G8.2 |  |
|  |  |  |  |  |  |  |  |  |  |  |  |  |  |  |  |  |  |  |  |  |  |  |  |  |  |  |  |  |  |  |  |  |  |  |  |  |  | *linc-92* | Long Intervening Non-Coding RNA |
|  |  |  |  |  |  |  |  |  |  |  |  |  |  |  |  |  |  |  |  |  |  |  |  |  |  |  |  |  |  |  |  |  |  |  |  |  |  | B0302.4 |  |
|  |  |  |  |  |  |  |  |  |  |  |  |  |  |  |  |  |  |  |  |  |  |  |  |  |  |  |  |  |  |  |  |  |  |  |  |  |  | H25P06.4 |  |
|  |  |  |  |  |  |  |  |  |  |  |  |  |  |  |  |  |  |  |  |  |  |  |  |  |  |  |  |  |  |  |  |  |  |  |  |  |  | W09C2.7 |  |
|  |  |  |  |  |  |  |  |  |  |  |  |  |  |  |  |  |  |  |  |  |  |  |  |  |  |  |  |  |  |  |  |  |  |  |  |  |  | ZK1290.10 |  |
|  |  |  |  |  |  |  |  |  |  |  |  |  |  |  |  |  |  |  |  |  |  |  |  |  |  |  |  |  |  |  |  |  |  |  |  |  |  | K10C9.12 |  |
|  |  |  |  |  |  |  |  |  |  |  |  |  |  |  |  |  |  |  |  |  |  |  |  |  |  |  |  |  |  |  |  |  |  |  |  |  |  | D1005.5 |  |
|  |  |  |  |  |  |  |  |  |  |  |  |  |  |  |  |  |  |  |  |  |  |  |  |  |  |  |  |  |  |  |  |  |  |  |  |  |  | T14F9.7 |  |
|  |  |  |  |  |  |  |  |  |  |  |  |  |  |  |  |  |  |  |  |  |  |  |  |  |  |  |  |  |  |  |  |  |  |  |  |  |  | K05G3.1 |  |
|  |  |  |  |  |  |  |  |  |  |  |  |  |  |  |  |  |  |  |  |  |  |  |  |  |  |  |  |  |  |  |  |  |  |  |  |  |  | W03D8.2 |  |
|  |  |  |  |  |  |  |  |  |  |  |  |  |  |  |  |  |  |  |  |  |  |  |  |  |  |  |  |  |  |  |  |  |  |  |  |  |  | *nhr-161* | Nuclear Hormone Receptor family |
|  |  |  |  |  |  |  |  |  |  |  |  |  |  |  |  |  |  |  |  |  |  |  |  |  |  |  |  |  |  |  |  |  |  |  |  |  |  | *clec-260* | C-type LECtin |
|  |  |  |  |  |  |  |  |  |  |  |  |  |  |  |  |  |  |  |  |  |  |  |  |  |  |  |  |  |  |  |  |  |  |  |  |  |  | K02F6.1 |  |
|  |  |  |  |  |  |  |  |  |  |  |  |  |  |  |  |  |  |  |  |  |  |  |  |  |  |  |  |  |  |  |  |  |  |  |  |  |  | *copd-1* | COat Protein complex 1, Delta subunit |
|  |  |  |  |  |  |  |  |  |  |  |  |  |  |  |  |  |  |  |  |  |  |  |  |  |  |  |  |  |  |  |  |  |  |  |  |  |  | *clec-202* | C-type LECtin |
|  |  |  |  |  |  |  |  |  |  |  |  |  |  |  |  |  |  |  |  |  |  |  |  |  |  |  |  |  |  |  |  |  |  |  |  |  |  | Y46H3A.5 |  |
|  |  |  |  |  |  |  |  |  |  |  |  |  |  |  |  |  |  |  |  |  |  |  |  |  |  |  |  |  |  |  |  |  |  |  |  |  |  | *glc-2* | Glutamate-gated ChLoride channel |
|  |  |  |  |  |  |  |  |  |  |  |  |  |  |  |  |  |  |  |  |  |  |  |  |  |  |  |  |  |  |  |  |  |  |  |  |  |  | T24E12.5 |  |
|  |  |  |  |  |  |  |  |  |  |  |  |  |  |  |  |  |  |  |  |  |  |  |  |  |  |  |  |  |  |  |  |  |  |  |  |  |  | *fbxc-37* | F-box C protein |
|  |  |  |  |  |  |  |  |  |  |  |  |  |  |  |  |  |  |  |  |  |  |  |  |  |  |  |  |  |  |  |  |  |  |  |  |  |  | K07E3.9 |  |
|  |  |  |  |  |  |  |  |  |  |  |  |  |  |  |  |  |  |  |  |  |  |  |  |  |  |  |  |  |  |  |  |  |  |  |  |  |  | C06A8.6 |  |
|  |  |  |  |  |  |  |  |  |  |  |  |  |  |  |  |  |  |  |  |  |  |  |  |  |  |  |  |  |  |  |  |  |  |  |  |  |  | ZK218.7 |  |

### Phenotypes enriched

none found

### Anatomy terms enriched

none found

### GO terms enriched

none found

### Expression clusters enriched

none found

### Motifs enriched

|  |  |  |  |  |  |
| --- | --- | --- | --- | --- | --- |
| **Motif** | **Logo** | **Possible orthologs** | **Number of motifs in cluster** | **Enrichment** | **FDR corrected p** |
| pTH5267 |  | hlh-32 (-0.51) hlh-16 | 61 | 1.74 | 0.00028 |
| pTH5074 |  | hlh-32 (-0.51) hlh-15 hlh-8 ngn-1 lin-31 | 106 | 1.33 | 0.00033 |
| pTH5099 |  | lin-32 (-0.56) hlh-1 hlh-15 hlh-11 | 99 | 1.37 | 0.00044 |
| pTH9391 |  | atf-2 ces-2 Y51H4A.4 | 123 | 1.21 | 0.00050 |
| HOXA2\_1 |  | ceh-2 (0.86) ceh-45 (0.69) lin-39 (-0.68) pha-2 (0.61) ceh-53 (0.56) ceh-18 alr-1 ceh-12 lim-6 ceh-14 mls-2 | 67 | 1.61 | 0.00072 |
| Titf1\_1722 |  | ceh-24 dsc-1 | 112 | 1.27 | 0.00085 |
| MA0066.1 |  | nhr-71 (0.82) nhr-6 nhr-43 | 107 | 1.30 | 0.00085 |
| pTH5159 |  | mxl-1 (-0.61) mxl-2 (-0.53) aha-1 | 114 | 1.25 | 0.00097 |
| pTH3997 |  | C04F5.9 | 66 | 1.61 | 0.00097 |
| FOXC1\_3 |  | lin-31 let-381 | 24 | 2.73 | 0.00120 |
| MITF\_f1 |  | hlh-30 irx-1 | 116 | 1.23 | 0.00160 |
| Hoxa3\_2783 |  | lin-39 (-0.68) | 34 | 2.16 | 0.00160 |
| pTH8916 |  | hmg-12 (-0.61) Y116A8C.22 | 51 | 1.75 | 0.00220 |
| pTH10034 |  | nhr-66 (0.67) | 84 | 1.42 | 0.00230 |
| pTH9323 |  | nhr-28 (0.82) odr-7 | 107 | 1.27 | 0.00250 |
| Ceh-22 |  | ceh-24 dsc-1 | 117 | 1.22 | 0.00260 |
| Meox1\_2310 |  | ceh-31 | 62 | 1.59 | 0.00270 |
| pTH10647 |  | nhr-232 | 22 | 2.69 | 0.00310 |
| MA0475.1 |  | lin-1 (-0.51) C24A1.2 | 46 | 1.79 | 0.00330 |
| MA0162.2 |  | ZC328.2 | 42 | 1.85 | 0.00400 |
| HLH4C\_da\_SANGER\_5\_FBgn0011277 |  | hlh-1 hlh-15 | 113 | 1.23 | 0.00400 |
| Hmx3\_3490 |  | ceh-9 | 125 | 1.16 | 0.00400 |
| pTH9059 |  | ztf-28 ces-1 | 119 | 1.20 | 0.00410 |
| Meis2\_1 |  | ceh-32 ces-1 | 41 | 1.86 | 0.00450 |
| pTH6108 |  | lin-31 let-381 | 106 | 1.26 | 0.00470 |
| PDX1\_do |  | lin-39 (-0.68) | 72 | 1.47 | 0.00480 |
| V$HEN1\_01 |  | hlh-15 | 102 | 1.28 | 0.00500 |
| TBX20\_1 |  | mab-9 tbx-38 tbx-39 tbx-43 | 77 | 1.43 | 0.00500 |
| MEIS3\_2 |  | ceh-32 | 42 | 1.83 | 0.00500 |
| Gsh2\_3990 |  | ceh-31 | 34 | 2.02 | 0.00510 |
| amos\_da\_SANGER\_10\_FBgn0003270 |  | hlh-15 | 114 | 1.22 | 0.00520 |
| tgo\_ss\_SANGER\_5\_FBgn0015014 |  | aha-1 | 60 | 1.57 | 0.00550 |
| FOXO1\_1 |  | fkh-8 daf-16 lin-31 fkh-10 let-381 | 111 | 1.23 | 0.00560 |
| pTH9137 |  | nhr-65 (0.87) | 34 | 2.01 | 0.00560 |
| Lbx2\_3869 |  | mls-2 | 72 | 1.46 | 0.00570 |
| klu\_SOLEXA\_5\_FBgn0013469 |  | ZC328.2 | 52 | 1.66 | 0.00580 |
| pTH5828 |  | nhr-84 (0.85) nhr-142 | 41 | 1.83 | 0.00620 |
| NR4A3\_f1 |  | nhr-6 nhr-2 Y67D8A.3 | 87 | 1.36 | 0.00650 |
| MA0483.1 |  | odd-1 | 49 | 1.68 | 0.00750 |
| V$FOXO1\_02 |  | daf-16 lin-31 | 75 | 1.42 | 0.00820 |
| Pou3f3\_3235 |  | ceh-6 | 24 | 2.35 | 0.00830 |
| pTH3120 |  | K11D2.4 | 89 | 1.33 | 0.00850 |
| pTH9216 |  | ceh-18 | 74 | 1.42 | 0.00950 |
| pTH6327 |  | dsc-1 | 114 | 1.20 | 0.00990 |
| MA0168.1 |  | ceh-19 (0.91) ceh-9 ceh-31 | 97 | 1.28 | 0.01000 |
| pTH10630 |  | lsy-27 | 116 | 1.19 | 0.01000 |
| Evx1\_3952 |  | ceh-53 (0.56) | 68 | 1.46 | 0.01000 |
| CrebA\_SANGER\_5\_FBgn0004396 |  | C27D6.4 | 37 | 1.85 | 0.01100 |
| Hoxa2\_3079 |  | lin-39 (-0.68) | 75 | 1.41 | 0.01100 |
| TCF7L1\_1 |  | pop-1 | 106 | 1.24 | 0.01100 |
| HepG2\_SRF\_HudsonAlpha |  | unc-120 | 40 | 1.79 | 0.01200 |
| PITX3\_1 |  | ceh-45 (0.69) ceh-53 (0.56) dve-1 alr-1 | 67 | 1.46 | 0.01200 |
| pTH3046 |  | Y116A8C.22 | 54 | 1.58 | 0.01200 |
| pTH9928 |  | jun-1 (0.63) | 68 | 1.45 | 0.01400 |
| PO3F2\_si |  | ceh-18 | 121 | 1.16 | 0.01400 |
| pTH9924 |  | nhr-46 (0.8) | 100 | 1.26 | 0.01500 |
| pTH10027 |  | M03D4.4 | 103 | 1.24 | 0.01600 |
| Zfp691\_0895 |  | F21A9.2 | 74 | 1.39 | 0.01700 |
| pTH6641 |  | lin-31 | 106 | 1.23 | 0.01700 |
| Dfd\_Cell\_FBgn0000439 |  | lin-39 (-0.68) php-3 | 41 | 1.73 | 0.01700 |
| V$GATA3\_03 |  | elt-1 (-0.62) nhr-10 | 55 | 1.54 | 0.01800 |
| SRP000217\_Klf4 |  | klf-1 | 35 | 1.84 | 0.01800 |
| pTH5164 |  | hlh-32 (-0.51) | 108 | 1.22 | 0.01800 |
| pTH9118 |  | eor-1 | 30 | 1.96 | 0.01900 |
| NR2F1\_4 |  | nhr-2 | 32 | 1.90 | 0.02000 |
| Lhx1\_2240 |  | lim-7 (0.58) | 100 | 1.25 | 0.02000 |
| Osr1\_3033 |  | odd-1 | 102 | 1.24 | 0.02200 |
| pTH9085 |  | nhr-42 (0.7) | 62 | 1.46 | 0.02200 |
| pTH9290 |  | ceh-18 | 34 | 1.83 | 0.02300 |
| Gm4881 |  | lin-1 (-0.51) | 21 | 2.30 | 0.02400 |
| pTH9381 |  | ceh-18 | 10 | 3.84 | 0.02400 |
| Etv3 |  | lin-1 (-0.51) | 20 | 2.35 | 0.02500 |
| pTH2193 |  | nhr-2 | 34 | 1.82 | 0.02500 |
| MA0467.1 |  | ceh-45 (0.69) | 75 | 1.37 | 0.02600 |
| Irx3\_0920 |  | irx-1 | 67 | 1.42 | 0.02600 |
| pTH9256 |  | ceh-18 | 57 | 1.49 | 0.02600 |
| TFEC\_1 |  | hlh-30 | 99 | 1.24 | 0.02700 |
| MA0037.2 |  | elt-1 (-0.62) | 86 | 1.30 | 0.02700 |
| F$MCM1\_01 |  | unc-120 | 116 | 1.17 | 0.02800 |
| pTH9261 |  | dmd-3 | 98 | 1.25 | 0.02800 |
| ELF1\_f1 |  | C24A1.2 | 38 | 1.72 | 0.02800 |
| pTH9885 |  | ceh-18 unc-86 | 27 | 1.99 | 0.02900 |
| CG7386\_F10-12\_SANGER\_5\_FBgn0035691 |  | F56D1.1 | 85 | 1.31 | 0.02900 |
| Nkx6-3\_3446 |  | cog-1 | 40 | 1.69 | 0.02900 |
| Foxj3\_1 |  | lin-31 | 89 | 1.29 | 0.03000 |
| V$CEBP\_01 |  | C48E7.11 | 94 | 1.26 | 0.03000 |
| pTH3751 |  | tbx-39 | 23 | 2.13 | 0.03100 |
| Mf26 |  | mef-2 (0.79) | 78 | 1.34 | 0.03100 |
| I$ABDB\_01 |  | ceh-24 | 34 | 1.79 | 0.03200 |
| I$DRI\_01 |  | cfi-1 | 101 | 1.23 | 0.03300 |
| V$XFD3\_01 |  | let-381 | 55 | 1.50 | 0.03300 |
| MAX\_1 |  | mxl-1 (-0.61) | 91 | 1.27 | 0.03300 |
| MSX1\_2 |  | lim-7 (0.58) ceh-1 | 87 | 1.29 | 0.03400 |
| Hr78\_SANGER\_5\_FBgn0015239 |  | nhr-213 (0.92) nhr-69 nhr-19 | 101 | 1.23 | 0.03400 |
| pTH5778 |  | egl-5 | 82 | 1.31 | 0.03400 |
| pTH5922 |  | ceh-24 | 104 | 1.21 | 0.03400 |
| pTH9326 |  | nhr-122 | 78 | 1.33 | 0.03400 |
| pTH6482 |  | ceh-19 (0.91) | 66 | 1.41 | 0.03500 |
| Hlx1\_2350 |  | ceh-24 | 58 | 1.46 | 0.03600 |
| pTH1292 |  | pzf-1 | 118 | 1.15 | 0.03600 |
| pTH10721 |  | ztf-9 | 61 | 1.44 | 0.03700 |
| pTH9135 |  | pop-1 | 98 | 1.24 | 0.03800 |
| pTH9300 |  | dmd-3 | 63 | 1.42 | 0.03900 |
| NR1D1\_f1 |  | nhr-118 | 89 | 1.27 | 0.04200 |
| EHF\_si |  | C24A1.2 | 49 | 1.53 | 0.04300 |
| pTH9188 |  | dmd-5 | 109 | 1.19 | 0.04400 |
| pTH9365 |  | ceh-18 | 116 | 1.16 | 0.04500 |
| IRX2\_1 |  | irx-1 | 122 | 1.13 | 0.04500 |
| pTH9297 |  | ceh-18 | 58 | 1.45 | 0.04500 |
| Alx4\_1744 |  | eyg-1 (0.62) alr-1 | 83 | 1.30 | 0.04600 |
| Prrx2\_3072 |  | ceh-45 (0.69) | 89 | 1.27 | 0.04600 |
| PAX1\_1 |  | pax-2 | 94 | 1.24 | 0.04800 |
| PRRX1\_f1 |  | alr-1 | 104 | 1.20 | 0.04800 |
| Cart1\_1275 |  | alr-1 | 81 | 1.30 | 0.05000 |

### Correlated (and anti-correlated) transcription factors

|  |  |
| --- | --- |
| **Transcription factor** | **Correlation** |
| nhr-213 | 0.92 |
| nhr-21 | 0.92 |
| ceh-19 | 0.91 |
| nhr-45 | 0.89 |
| nhr-54 | 0.89 |
| nhr-65 | 0.87 |
| ceh-2 | 0.86 |
| nhr-84 | 0.85 |
| nhr-62 | 0.84 |
| nhr-134 | 0.84 |
| nhr-238 | 0.83 |
| nhr-71 | 0.82 |
| nhr-116 | 0.82 |
| nhr-203 | 0.82 |
| nhr-49 | 0.82 |
| nhr-195 | 0.82 |
| nhr-28 | 0.82 |
| nhr-117 | 0.81 |
| gmeb-2 | 0.81 |
| ceh-22 | 0.81 |
| pax-1 | 0.80 |
| nhr-46 | 0.80 |
| nhr-182 | 0.80 |
| nhr-125 | 0.80 |
| nhr-211 | 0.80 |
| cnd-1 | -0.50 |
| C01F6.9 | -0.51 |
| cebp-2 | -0.51 |
| hlh-32 | -0.51 |
| lin-1 | -0.51 |
| tlp-1 | -0.52 |
| unc-39 | -0.52 |
| ceh-20 | -0.52 |
| nhr-172 | -0.53 |
| mxl-2 | -0.53 |
| tbx-31 | -0.53 |
| elk-2 | -0.54 |
| tbx-35 | -0.54 |
| Y56A3A.18 | -0.54 |
| lin-32 | -0.56 |
| unc-62 | -0.59 |
| hmg-12 | -0.61 |
| mxl-1 | -0.61 |
| elt-1 | -0.62 |
| F21D5.9 | -0.64 |
| ceh-13 | -0.66 |
| lin-39 | -0.68 |
| mab-5 | -0.69 |
| hmg-11 | -0.69 |
| bar-1 | -0.70 |

### ChIP peaks enriched

none found
